# Supplementary material for: How prior preferences determine decision-making frames and biases in the human brain
Source: eLife. 2016 Nov 19;5:e20317. doi: 10.7554/eLife.20317 (PMC5132340; doi:10.7554/eLife.20317)
Supplement: Supplementary file 1. — Regions are listed that survived voxel-based thresholding of p<0.001 uncorrected, and whole-brain 991 cluster-level FWE correction. [x, y, z] coordinates refer to the Montreal Neurological Institute (MNI) space. DOI: http://dx.doi.org/10.7554/eLife.20317.008 [file elife-20317-supp1.docx]

**SUPPLEMENTARY FILE 1**

| V_IT_(def)-V_IT_(alt) | | | | | | | | | | | |
| --- | --- | --- | --- | --- | --- | --- | --- | --- | --- | --- | --- |
| **Cluster** | **Cluster size** | **peak side** | **x** | **y** | | **z** | | **Z score** | | **T value** | **Cluster p value** |
| vmPFC | 1262 | R | 2 | 40 | | 10 | | 4.85 | | 6.51 | 9.10^-10^ |
| Ventral striatum | 675 | L | -18 | 4 | | 10 | | 4.74 | | 6.28 | 3.10^-6^ |
|  | | | | | | | | | | | |
| V_IT_(ch)-V_IT_(unch) | | | | | | | | | | | |
| **Cluster** | **Cluster size** | **peak side** | **x** | **y** | | **z** | | **Z score** | | **T value** | **Cluster p value** |
| Rolandic operculum | 342 | L | -56 | 2 | | 8 | | 4.92 | | 6.66 | 0.004 |
| Inferior parietal lobule | 367 | R | 64 | -24 | | 40 | | 4.71 | | 6.21 | 0.003 |
|  | 368 | L | -66 | -38 | | 36 | | 4.56 | | 5.90 | 0.003 |
| Angular gyrus | 208 | R | -24 | -44 | | 28 | | 4.52 | | 5.82 | 0.035 |
|  | | | | | | | | | | | |
| V_IT_(unch)-V_IT_(ch) | | | | | | | | | | | |
| **Cluster** | **Cluster size** | **peak side** | **x** | | **y** | | **z** | | **Z score** | **T value** | **Cluster p value** |
| Middle occipital gyrus | 4901 | R | 32 | | -80 | | 18 | | 6.18 | 10.08 | <1.10^-11^ |
| dACC | 2202 | R | 10 | | 26 | | 32 | | 5.74 | 9.66 | 2.10^-11^ |
| Anterior insula | 1774 | L | -28 | | 26 | | 4 | | 5.83 | 8.99 | 6.10^-10^ |
| Middle occipital gyrus | 5289 | L | -28 | | -84 | | 20 | | 5.74 | 8.73 | 6.10^-4^ |
| Anterior insula | 876 | R | 30 | | 26 | | -2 | | 4.84 | 6.48 | 4.10^-6^ |

**Table S1 – Activation list for decision value coding in the pre-choice and post-choice frames (GLM 3).**

Regions are listed that survived voxel-based thresholding of p<0.001 uncorrected, and whole-brain cluster-level FWE correction. [x, y, z] coordinates refer to the Montreal Neurological Institute (MNI) space.
